# The progression of intracerebral hemorrhage (ICH) is related to the expression of integrin Β1 (ITGB1)

**DOI:** 10.1186/s41016-021-00234-4

**Published:** 2021-03-01

**Authors:** Hai-Yang Ma, Yan Xu, Chun-You Qiao, Yi Peng, Qi Ding, Li-Zhong Wang, Jun-Fei Yan, Yuan Hou, Fei Di

**Affiliations:** 1 Department of Neurosurgery, Beijing Tiantan Hospital, Capital Medical University, Beijing, 100160 China; 2 Department of Endocrinology, ZhangJiakou First Hospital, Zhangjiakou, 075041 China; 3 Department of Neurosurgery, ZhangJiakou First Hospital, Zhangjiakou, 075041 China

**Keywords:** Biomarker, Immunohistochemical staining, Inflammation, Integrins, Intracerebral hemorrhage, Western blotting

## Abstract

**Background:**

Intracerebral hemorrhage (ICH) is fatal and detrimental to quality of life. Clinically, options for monitoring are often limited, potentially missing subtle neurological changes. Integrin β 1 (ITGB1) and β 3 (ITGB3) are the main components of integrin family receptors, which regulate the formation and stability of blood vessels. This study explored the relationship between the expression of ITGB1 and ITGB3 in intracerebral hemorrhage (ICH) to analyze their functional and clinical relevance.

**Methods:**

The expression of ITGB1 and ITGB3 in ICH was accomplished by immunohistochemical (IHC) staining and western blotting (WB) analysis, respectively.

**Results:**

Furthermore, the results demonstrated that ITGB1 was expressed in ICH tissues, but ITGB3 was not expressed in ICH tissues.

**Conclusions:**

In summary, the clinical progression of ICH was related to the expression of ITGB1. ITGB1 may be a potential biomarker and contribute to the treatment of ICH.

## Background

Intracerebral hemorrhage (ICH) is a critical type of cerebrovascular disease with a high incidence [1]. Although the treatment strategy of ICH in the acute phase has become more and more standardized, it is still limited to hematoma clearance and symptomatic supportive treatment [2, 3]. At present, there is no effective treatment for the loss of neurological function caused by cerebral hemorrhage, so the mortality and disability rate are still high in the past 10 years [4]. It is known that the injury mechanism of intracerebral hemorrhage indicated that the composition, structure, and distribution of extracellular matrix (ECM) have changed [5]. In addition, the destruction of blood-brain barrier (BBB) and the formation of brain edema are closely related to ECM [6, 7]. Previous studies have confirmed that ECM is involved in the formation, development, repair, and regeneration of embryos and various tissues and organs [8, 9].

The integrins are a superfamily of cell adhesion receptors that bind to ECM ligands, cell-surface ligands, and soluble ligands [10]. ECM participates in the interaction between cells through the mediation of integrins [11]. Once the nervous system is injured, integrins can affect nerve regeneration by promoting the survival of neurons, regulating the length of nerve axons, and participating in the directional migration and differentiation of nerve cells [12]. Moreover, integrins are involved in almost every stage of cancer progression from primary tumor to metastasis through their role in signaling molecules, mechanical transducers, and key components of cell migration mechanisms [13]. Integrin β 1 (ITGB1) and β 3 (ITGB3) are the main components of integrin family receptors, which regulate the formation and stability of blood vessels [14–16]. However, whether the injury of ICH leads to the alteration of ITGB1 and ITGB3 has not been confirmed.

The purpose of this study was to explore the mechanism of tissue and neural function recovery by analyzing the expression of ITGB1 and ITGB3 in ICH patients. This would provide new clues and ideas for the development and treatment strategies of basic and clinical exploration of ICH.

## Methods

### Tissue slide collection

The tissues of 12 patients with ICH and the matched normal tissue slides were collected. Meanwhile, pathological characteristics of these samples were obtained, including age, smoking history, drinking history, diabetes history, systolic blood pressure, diastolic blood pressure, fasting blood glucose, and lipid levels. All patients in this study signed informed consents.

### Immunohistochemical (IHC) staining

Firstly, the tissue slides were placed at 65 °C for 30 min, then dewaxed with xylene, and washed with alcohol. Then, the tissue slides were repaired by citrate buffer, cooled to room temperature, and soaked in 1 × PBST buffer (1 × PBS + 0.1% Tween 20) for 5 min. Next, the tissue slides were sealed with 3% H_2_O_2_ and 5% serum for 15 min, respectively. Following, they were incubated with anti-ITGB1 antibody (1:200, abcam, Cat # ab8991) and anti-ITGB3 antibody (1:200, abcam, Cat # ab119992) overnight at 4 °C, respectively. The slides were washed with 1 × PBST buffer solution for 5 min/3 times, and then, the secondary antibody HRP Goat Anti-Rabbit IgG (1:200, abcam, Cat #ab111909) was added and placed at 37 °C for 1 h. After washing the slides at the end of the secondary antibody reaction with 1 × PBST buffer, the slides were dyed for 5 min with DAB solution, and then staining was terminated by washing with H_2_O. After that, it was re-dyed with hematoxylin for 15 s and finally sealed with neutral gum.

IHC scores were determined by staining percentage scores (classified as 1 (1–24%), 2 (25–49%), 3 (50–74%), and 4 (75–100%)) and staining intensity scores (scored as 0, signal less color; 1, brown; 2, light yellow; and 3, dark brown). To distinguish between high and low expression, the median was selected as the cut-off value to reduce the impact of outliers. All tissue microarray chips were pictured with microscopic and viewed with Image Scope and Case Viewer.

### Animal model construction

In this study, 12 clean grade Sprague-Dawley (SD) rats (250–3008, 8–10 weeks old), half male and half female, were reared in the SPF animal room. The animals were kept in a cage with alternating light and dark for 12 h, keeping the feeding temperature at 20 °C and humidity at 50–60%. The experiment was divided into two parts: in the first part, 12 rats were numbered one by one and randomly divided into the sham operation group (*n* = 3) and the cerebral hemorrhage group (*n* = 9). The animal model of the cerebral hemorrhage group was injected with collagenase (0.2 U/μL VII collagenase) prepared by 2.5 μL normal saline, and the sham operation group was injected with 2.5 μL normal saline. The rats were killed at 4 days, 7 days, and 21 days after the establishment of the model, respectively. Three rats were randomly selected at each time point, and one in the control group was killed. Then, western blotting (WB) was used to detect the expression of ITGB1 and ITGB3 in the brain tissue of rats with hemorrhagic stroke.

### Western blotting (WB) analysis

The experiment was divided into the control (CON) and ICH groups. The rats in the ICH group were sacrificed 4 days, 7 days, and 21 days after modeling, and the expressions of ITGB1 and ITGB3 in the ICH tissues of the rats with hemorrhagic stroke were detected by WB. Firstly, the proteins were extracted with cell lysate and detected by BCA protein detection kit (HyClone-Pierce). The 10-μg protein was separated by SDS-PAGE (Invitrogen) and transferred to the PVDF membrane, then sealed at room temperature for 1 h with TBST solution. After that, the membrane was first incubated with primary antibodies (ITGB1, 1:1000, abcam, Cat # ab8991; ITGB3, abcam, Cat # 1:1000, ab119992; GAPDH, 1:3000, Bioworld, AP0063) at 37 °C for 2 h. Following, the membrane was incubated with HRP-conjugated (goat anti-rabbit, 1:3000, Beyotime, Cat # A0208; goat anti-mouse, 1:3000, Beyotime, Cat # A0216) at room temperature for 1 h. Finally, Millipore Immobilon Western Chemiluminescent HRP Substrate kit (Millipore, Cat # RPN2232) was used for color rendering and Chemiluminescent imager (GE, Cat # AI600) observation.

## Results

### Comparison of general clinical data of patients with stroke

**Fig. 1:**
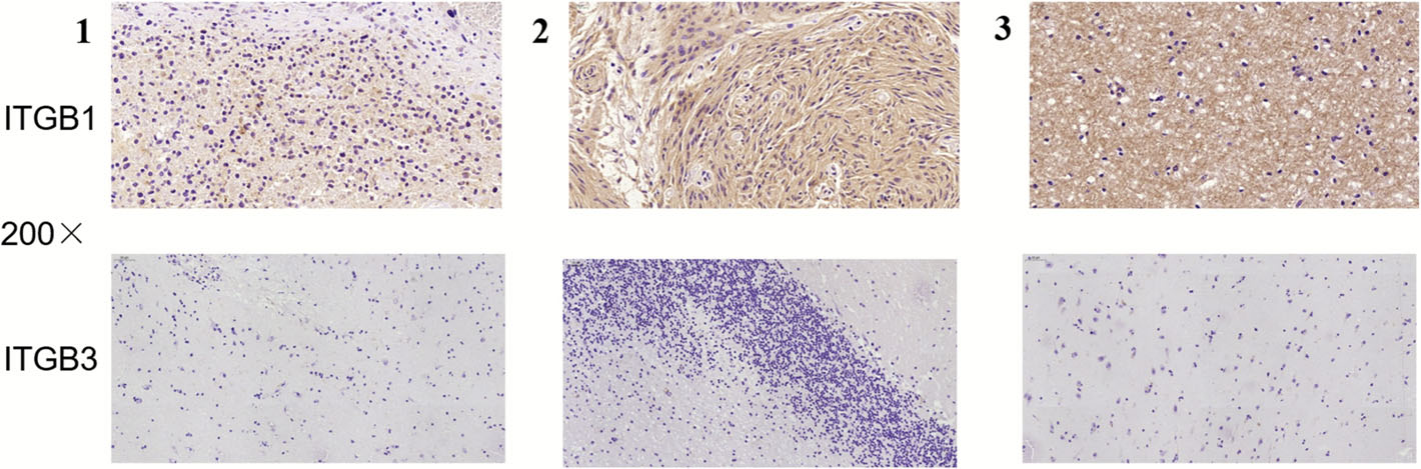
The expression of ITGB1 and ITGB3 in ICH was accomplished by IHC staining. The magnification is × 200

**Fig. 2:**
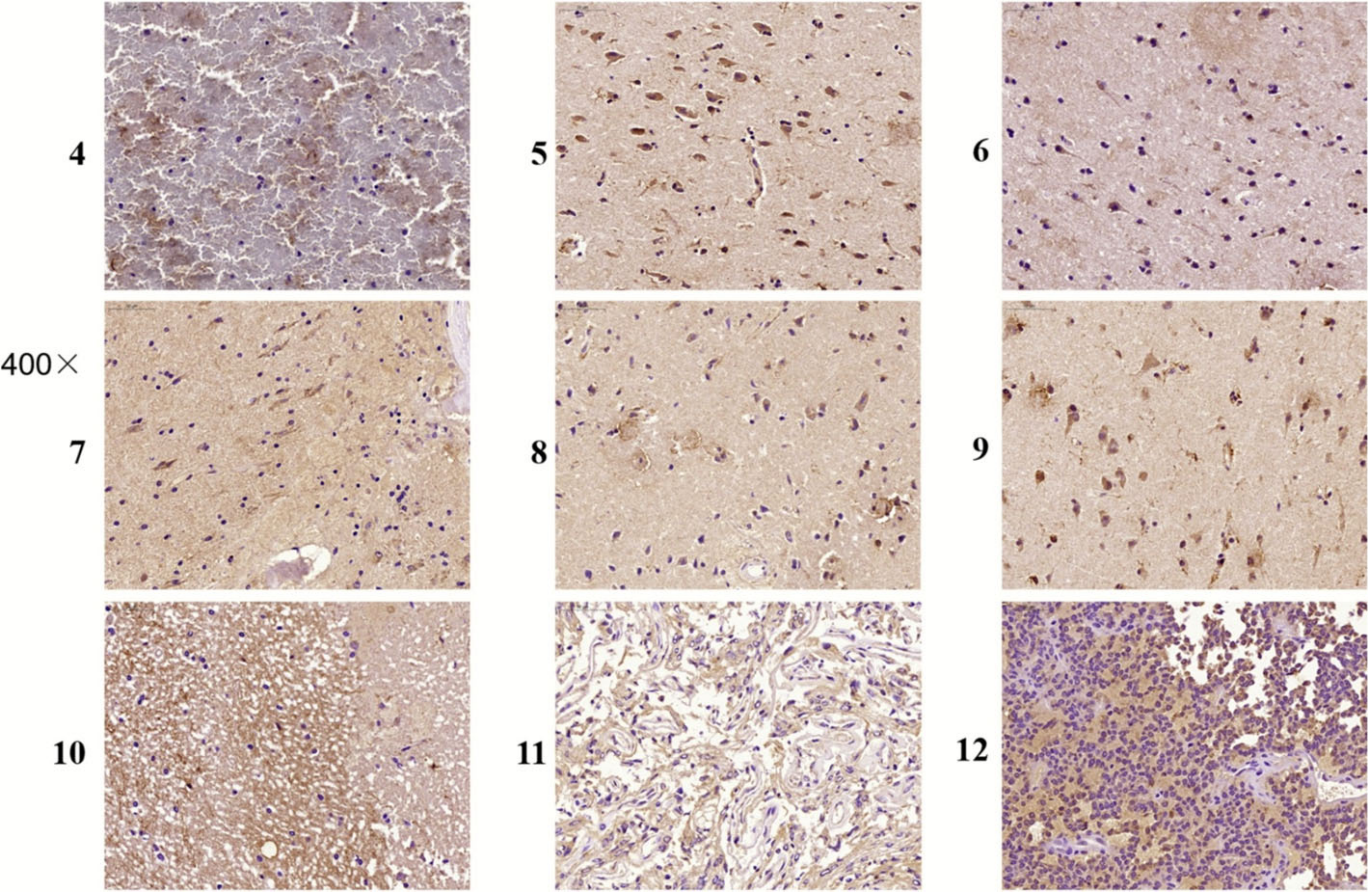
The expression of ITGB1 in ICH was detected by IHC staining

**Fig. 3:**
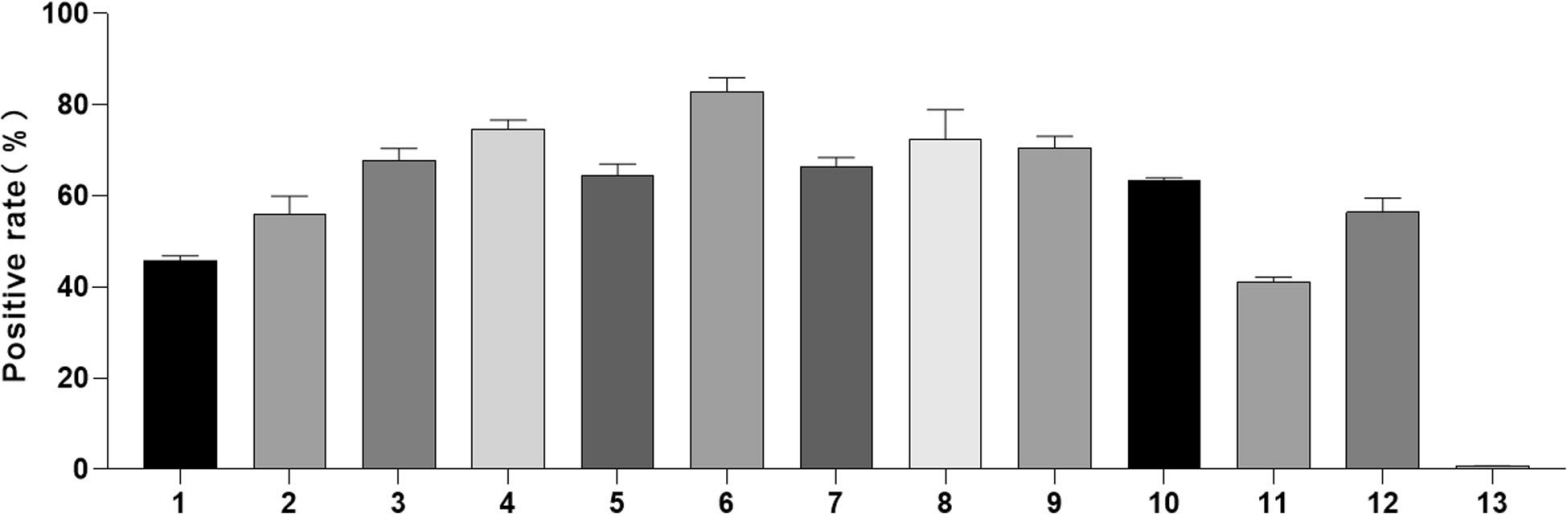
The expression of ITGB1 (positive cell staining rate) in 12 tissue slides was showed

The expression of ITGB1 and ITGB3 in tissue samples of patients with ICH was detected by IHC staining. As illustrated in Fig. 1, a positive cell line exhibited increased staining intensity for ITGB1. By contrast, ITGB3 was not detected in all samples. Moreover, Fig. 2 suggests that ITGB1 was expressed in different parts of ICH tissues. In addition, the positive rate of ITGB1 in ICH patient tissue samples is illustrated in Fig. 3. Accordingly, the expression of ITGB1 was relatively high in ICH tissues.

### Differences of ITGB1 and ITGB3 protein expression in animal models

**Fig. 4:**
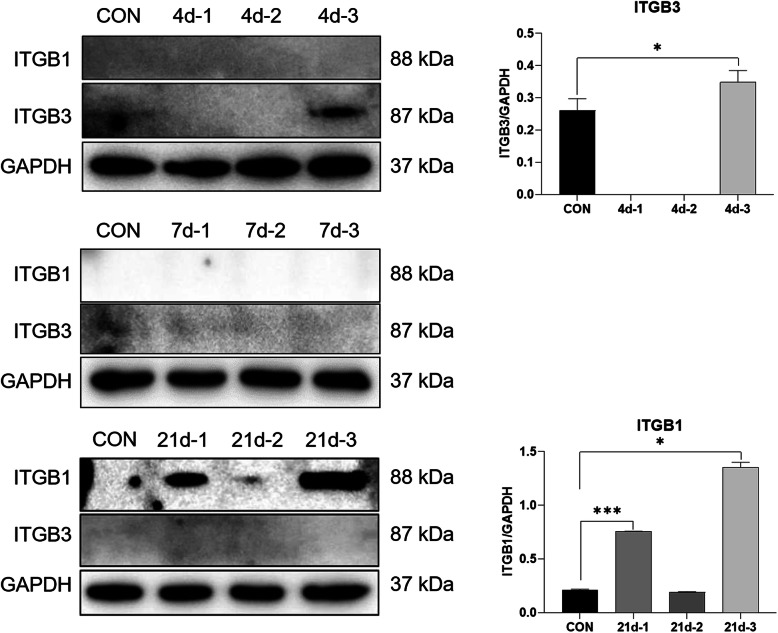
Differences of ITGB1 and ITGB3 protein expression in animal models at days 4, 7, and 21

The alteration of ITGB1 and ITGB3 expression in cerebral tissue of rats with hemorrhagic stroke was detected by WB. Compared with the control group, the protein expression level of ITGB3 in the brain tissue of the no. 3 rats in the ICH group increased significantly 4 days after the establishment of the model (*p* < 0.05). After 21 days, ITGB1 protein expression levels of the no. 1 and no. 3 rats in the ICH group were significantly increased (*p *< 0.05), whereas ITGB3 protein was not detected after 7 days (Fig. 4).

## Discussion

ICH remains a cause of significant morbidity and mortality and is associated with severe long-term disability [2]. In addition, its incidence is 24.6 per 100,000 person-years, and the related incidence is increasing as the population ages [17]. Despite this, ICH is the last form of stroke without specific therapy. Treatment of ICH ranges from best medical therapy to approaches involving several different surgical techniques, most of which are at different levels of experimental state [18]. A lack of definitive evidence-based recommendations to guide the care of patients with ICH has led to significant heterogeneity in current clinical practice.

The ECM, a non-cellular 3D macromolecular network composed of diverse fibrous ECM proteins, proteoglycans, and glycoproteins, not only provides a physical scaffold to structure the 3D microenvironment but also signals a variety of cellular responses [19, 20]. In particular, ECM-derived signals are transported to the cytoplasm through integrins that directly recognize components of the ECM, resulting in cytological alterations [6, 11]. Therefore, the stimulation of ECM protein-derived signals by integrins makes it possible to accurately regulate the specificity of cells.

ICH causes inflammation characterized by leukocyte recruitment and elevated levels of cytokines [21]. Specific leukocyte populations, including neutrophils, T cells, and inflammatory monocytes, promote secondary injury in intracerebral hemorrhage models [22]. A previous study demonstrated that integrin complex plays a significant role in cellular interactions with interstitial collagen that are involved in matrix remodeling such as is seen during morphogenesis and wound healing [23]. Hammond et al. suggested that blocking the function of α-4 integrin led to the decrease of leukocyte recruitment and the improvement of motor function after ICH [24]. Dardiotis et al. found that genetic polymorphism in the ITGAV and ITGB8 that may alter the structure or function of integrins may render individuals more susceptible to ICH [25]. At the present study, we indicated that ITGB1 was expressed in ICH tissues, but ITGB3 was not detectable in ICH tissues. The clinical progress of ICH is related to the expression of ITGB1, which was also proved by the animal model.

## Conclusions

Our results indicate that the clinical progression of ICH is related to the expression of ITGB1, which may be a potential biomarker of ICH. This would provide new clues and ideas for the development and treatment strategies of ICH.

## Data Availability

The datasets used and/or analyzed during the current study are available from the corresponding author on reasonable request.

## Abbreviations

BBB: Blood-brain barrier
CON: Control
DMEM: Dulbecco’s modified Eagle’s medium
ECM: Extracellular matrix
FBS: Fetal bovine serum
ICH: Intracerebral hemorrhage
IHC: Immunohistochemical
ITGB: Integrin β
SD: Sprague-Dawley
WB: Western blotting
